# IgG Subclass and Heavy Chain Domains Contribute to Binding and Protection by mAbs to the Poly γ-D-glutamic Acid Capsular Antigen of *Bacillus anthracis*

**DOI:** 10.1371/journal.ppat.1003306

**Published:** 2013-04-18

**Authors:** Maria Hovenden, Mark A. Hubbard, David P. AuCoin, Peter Thorkildson, Dana E. Reed, William H. Welch, C. Rick Lyons, Julie A. Lovchik, Thomas R. Kozel

**Affiliations:** 1 Department of Microbiology and Immunology, University of Nevada School of Medicine, Reno, Nevada, United States of America; 2 Cell and Molecular Biology Graduate Program, University of Nevada School of Medicine, Reno, Nevada, United States of America; 3 Department of Biochemistry and Molecular Biology, University of Nevada School of Medicine, Reno, Nevada, United States of America; 4 Department of Internal Medicine, University of New Mexico Health Sciences Center, Albuquerque, New Mexico, United States of America; Yale University School of Medicine, United States of America

## Abstract

Bacterial capsules are common targets for antibody-mediated immunity. The capsule of *Bacillus anthracis* is unusual among capsules because it is composed of a polymer of poly-γ-d-glutamic acid (γdPGA). We previously generated murine IgG3 monoclonal antibodies (mAbs) to γdPGA that were protective in a murine model of pulmonary anthrax. IgG3 antibodies are characteristic of the murine response to polysaccharide antigens. The goal of the present study was to produce subclass switch variants of the γdPGA mAbs (IgG3→IgG1→IgG2b→IgG2a) and assess the contribution of subclass to antibody affinity and protection. Subclass switch antibodies had identical variable regions but differed in their heavy chains. The results showed that a switch from the protective IgG3 to IgG1, IgG2b or IgG2a was accompanied by i) a loss of protective activity ii) a change in mAb binding to the capsular matrix, and iii) a loss of affinity. These results identify a role for the heavy chain constant region in mAb binding. Hybrid mAbs were constructed in which the CH1, CH2 or CH3 heavy chain constant domains from a non-protective, low binding IgG2b mAb were swapped into the protective IgG3 mAb. The IgG3 mAb that contained the CH1 domain from IgG2b showed no loss of affinity or protection. In contrast, swapping the CH2 or CH3 domains from IgG2b into IgG3 produced a reduction in affinity and a loss of protection. These studies identify a role for the constant region of IgG heavy chains in affinity and protection against an encapsulated bacterial pathogen.

## Author Summary

The ability of an antibody to recognize and bind to its target is classically viewed as a function of the variable region of the molecule; this region distinguishes an antibody with one specificity from an antibody with a different specificity. We examined binding of antibodies to an outer coat of the biothreat *Bacillus anthracis* that is essential for bacterial virulence. We identified regions of the antibody constant region which contribute to antibody binding and the ability of the antibody to protect the host. These constant regions are distinct from the variable regions that directly mediate antibody binding. The results of the study have implications for i) understanding how antibodies function in protection against anthrax and possibly other diseases, ii) understanding how the host responds to a key bacterial virulence factor, iii) selection of antibodies that might be used to treat anthrax, and iv) design of vaccines to protect against anthrax.

## Introduction

*Bacillus anthracis*, the causative agent of anthrax, is a gram-positive, spore-forming bacterium [1]. Due to the ease of infection and high lethality, *B. anthracis* has been listed by the Centers for Disease Control and Prevention as one of the Category A agents of bioterrorism. Virulent strains of *B. anthracis* carry two large plasmids, pXO1 and pXO2, that encode genes needed for toxin production and capsule formation, respectively [2], [3]. Anthrax toxins are composed of protective antigen (PA) combined with lethal factor (LF) or edema factor (EF) to form active toxins [4], [5]. The polypeptide capsule is composed of poly-γ-d-glutamic acid (γdPGA) [6] and is both poorly immunogenic and antiphagocytic [7]–[9].

The current vaccine, anthrax vaccine adsorbed (AVA, Biothrax), is composed of aluminum hydroxide-adsorbed, formalin-treated, cell-free filtrate of a non-capsulated *B. anthracis* strain [10]. The active component of AVA is believed to be PA. However, there has been recent interest in targeting γdPGA as an addition to a vaccine [11]–[16]. Antibodies to γdPGA are opsonic [11], [12], [15], [17]. γdPGA-based immunity is attractive because such immunity would interdict the infection when the bacterial load is low and would prevent infection from reaching the stage where large amounts of toxin are formed. As a consequence, γdPGA-specific antibodies could exhibit potent synergy with toxin-targeted immunity [11].

We recently reported production of monoclonal antibodies (mAbs) specific for the γdPGA capsule [13], [14]. Passive immunization with murine mAbs of the IgG3 subclass was protective in a murine model of pulmonary anthrax [13], [14]. This was the first report of protection mediated by *B. anthracis* capsular antibodies.

The ability of capsular antibodies to protect is related to the level of antibody generated in response to immunization as well as qualitative factors such as affinity and effector functions. Antibody affinity for antigen is largely determined by the variable regions of the amino-terminal domains of the heavy and light chains, whereas the effector functions and the IgG subclass are determined by the constant region of the heavy chain. In the mouse, the IgG subclasses are IgG1, IgG2a, IgG2b and IgG3. The murine constant region of the IgG heavy chain consists of three domains, CH1, CH2, and CH3. The region between the CH1 and CH2 domains is called the hinge region and permits flexibility in the chain.

A successful active or passive immunization strategy that targets γdPGA will require an understanding of the roles of IgG subclass in protection. The overall aim of this study was to assess the contribution of the constant region of the murine IgG heavy chain to protection in a murine model of inhalational anthrax. The results showed that murine IgG3 is highly protective, but the IgG1, IgG2a and IgG2b subclasses are poorly or non-protective and have a markedly reduced affinity compared to IgG3 antibodies. Hybrids of the protective IgG3 antibody having CH1, CH2 or CH3 domains of the non-protective IgG2b antibody were constructed in an effort to better understand the contribution of heavy chain domains to protection and antibody affinity. The results showed that the CH2 domain and to a lesser extent the CH3 domain were major determinants of both antibody affinity and protection.

## Results

### Contribution of IgG subclass to protection

The IgG3 mAbs F24F2 and F26G3 were produced from mice immunized with γdPGA in combination with agonist mAbs to CD40 [13]. Full subclass-switch families (IgG3→IgG1→IgG2b→IgG2a) from the parental F24F2 and F26G3 IgG3 mAbs were generated using sequential sib selection [18]. Once all eight mAb-secreting hybridoma cell lines were obtained, total mRNA was isolated and used for cDNA synthesis and PCR amplification. Sequencing of PCR products verified that the heavy- and light-chain variable regions were identical in all four subclasses (data not shown), and the heavy chain sequences corresponded to those reported for murine IgG3, IgG1, IgG2b, and IgG2a [19].

**Figure 1:**
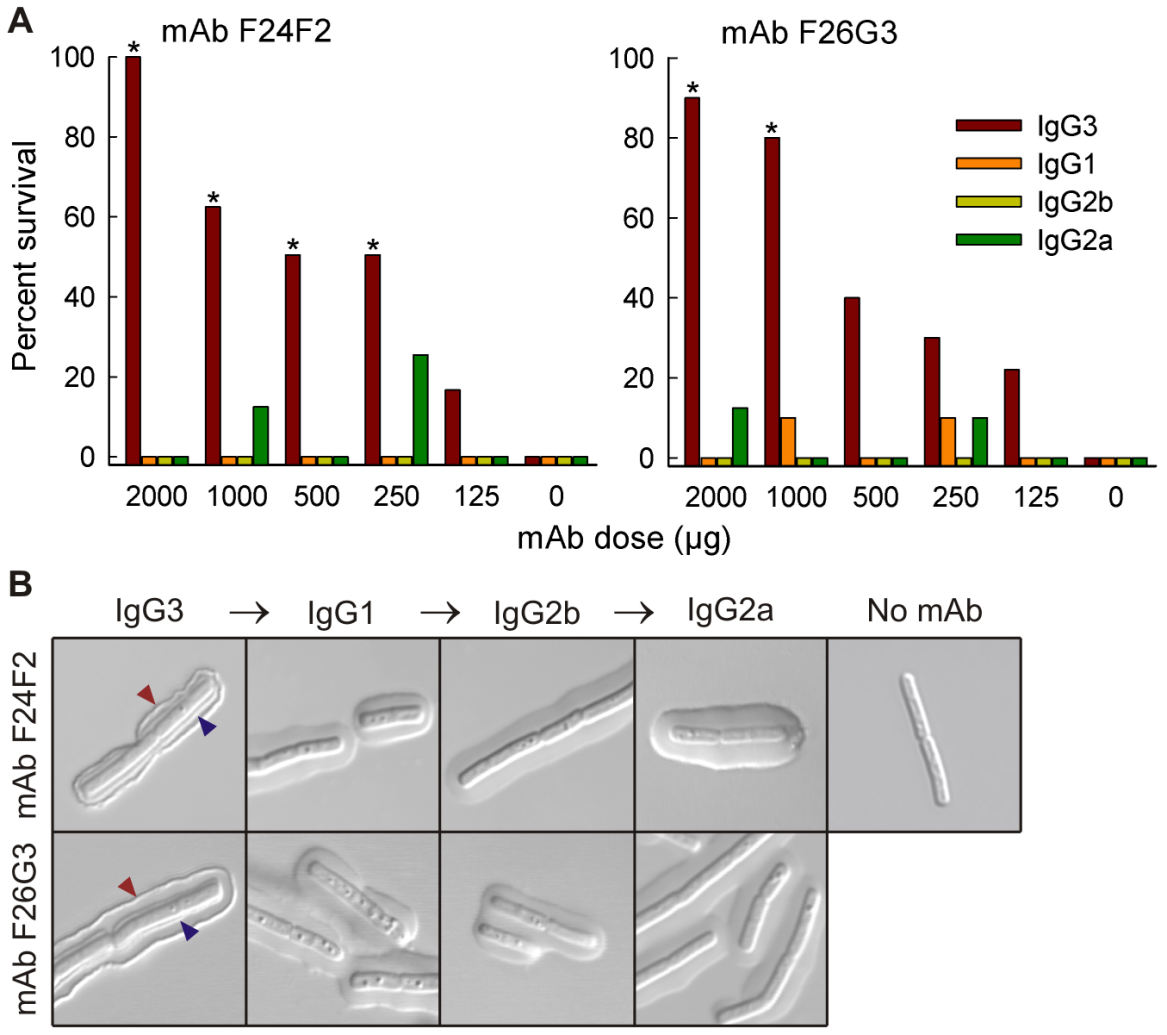
Protection and capsule reactivity of subclass switch families of mAbs F24F2 and F26G3. A – Protection afforded by passive immunization. Mice (n = 8–10 mice/group) were treated with the indicated dose of mAb and challenged 18 h later via the intratracheal route with 10 LD_50_ of *B. anthracis* spores (Ames). Statistical comparison of survival of mAb-treated mice vs. control mice given DPBS alone was done with Fisher exact test; * *P*<0.05. B – Capsule reaction produced by incubation of *B. anthracis* vegetative cells with mAbs of each subclass (75 µg/ml). Red arrow – outer rim produced on binding of IgG3 mAb; blue arrow – inner binding reaction of IgG3 near the cell wall.

An initial experiment determined protection afforded by the subclass switch variants in a murine model of pulmonary anthrax. Mice were treated with various doses of each mAb and challenged 18 h later with a lethal dose of *B. anthracis* (Ames) spores. The results from evaluation of subclass switch families of both mAbs F24F2 and F26G3 showed that only the IgG3 mAbs were protective; similar results were found with subclass switch families of both parental cell lines (Fig. 1A). Treatment with the IgG3 mAbs at doses between 125–2000 µg/mouse increased the overall percent survival of mice in a dose-dependent fashion. Treatment with IgG1, IgG2b, or IgG2a mAbs did not significantly increase the overall percent survival at any treatment dose (P>0.05).

The capsular quellung reaction is a means to evaluate interactions of antibodies with an intact capsule [20]. In previous studies of the opportunistic yeast *Cryptococcus neoformans*, we found that protective antibodies cross-link the capsule edge to produce an annular rim that is visible by differential interference contrast (DIC) microscopy [21]. Non-protective antibodies bind throughout the capsular matrix and form a puffy-type reaction that does not cross-link the edge. In this experiment, killed *B. anthracis* Ames bacilli were incubated with the subclass switch families of mAbs F24F2 and F26G3, and capsule reactions were analysed by DIC microscopy. The results (Fig. 1B) showed that the IgG3 mAbs produced capsule reactions with annular rim-type patterns at the outer edge of the capsule that are typical of protective mAbs in the *C. neoformans* system (Fig. 1B, red arrow). In addition, the IgG3 mAbs produced the previously described dual-capsule reaction [14] where there was a distinct reaction at a layer that was well beneath the capsule surface in the region of the cell wall (Fig. 1B, blue arrow). In contrast, switching from the IgG3 to IgG1, IgG2b, or IgG2a subclasses was accompanied by a switch to a puffy type of capsular reaction that was characteristic of non-protective mAbs in the *C. neoformans* system. Neither the outer rim pattern nor the pronounced inner layer of reactivity near the cell wall was observed with the switch variants. Similar results were found with subclass switch families derived from both parental mAbs.

**Figure 2:**
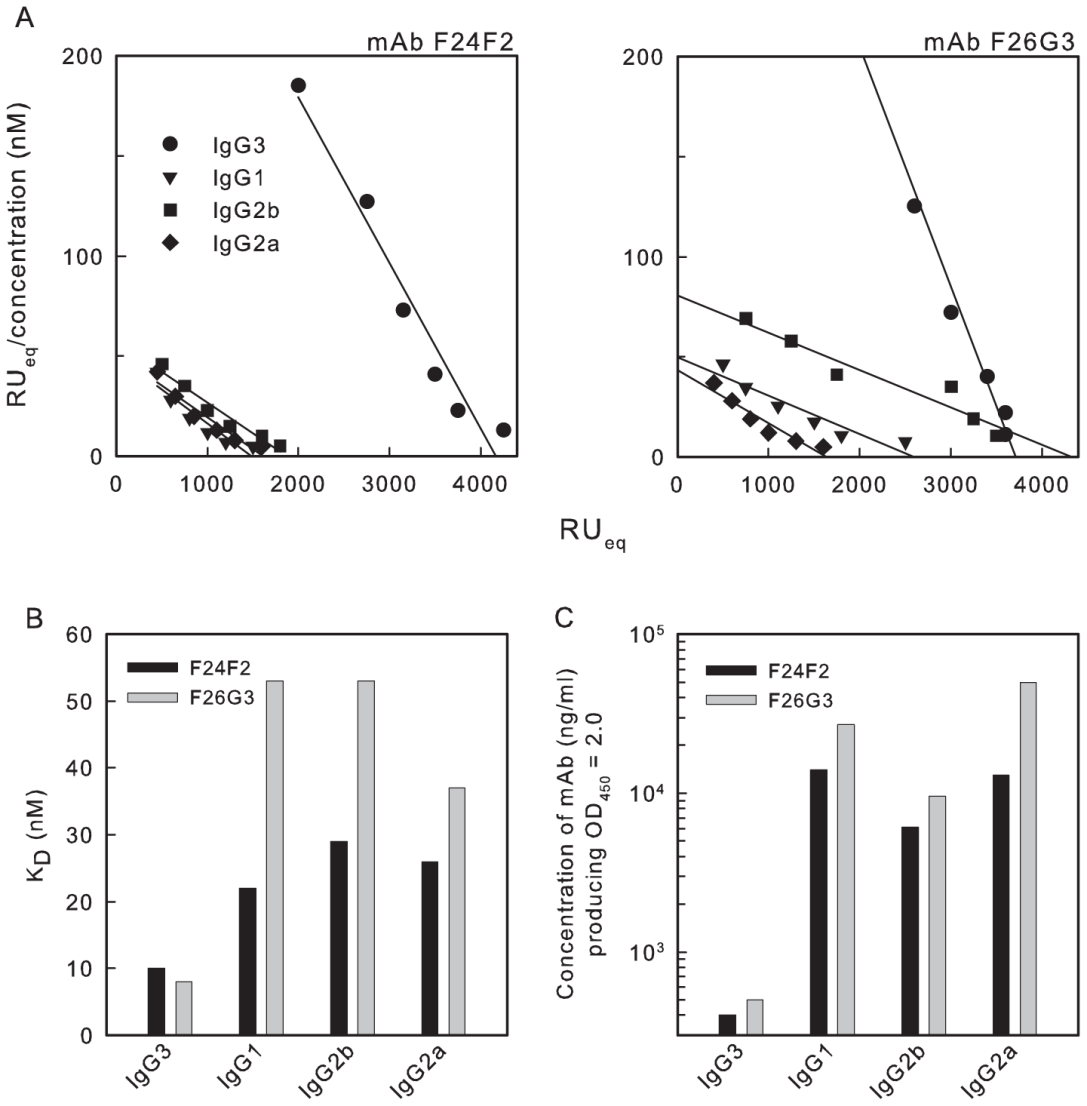
Binding of subclass switch families of mAbs F24F2 and F26G3 to γdPGA. A – Scatchard plots for determination of functional affinities from equilibrium binding. Binding was assessed by SPR using intact mAb and sensor chips coated with 25-mers of γdPGA. B – K_D_ for binding of each mAb shown in Panel A. K_A_ was determined from the slope of the regression lines in Panel A. K_D_ was calculated as 1/K_A_. C - Binding of each mAb as assessed by ELISA in which plates were coated with purified native γdPGA. Results are reported as the concentration of each mAb (ng/ml) that produced an OD_450_ = 2.0 in a standard ELISA format.

One explanation for the failure of the IgG1, IgG2b and IgG2a variants to both protect and produce the annular rim seen via DIC microscopy is the possibility that subclass switching was accompanied by a reduction in affinity such that the mAb could not cross-link the capsular matrix. As a consequence, the functional affinity of each member of the two subclass switch families was determined by surface plasmon resonance analysis. Functional affinity is defined as the overall strength of binding between a multivalent antigen and a multivalent antibody [22]–[24]. Equilibrium binding was assessed using sensor chips coated with a 25-mer of γdPGA. The results are shown in Fig. 2A as a Scatchard plot in which RU_eq_/concentration is plotted against RU_eq_. Fig. 2B provides a summary of the Scatchard analysis where the results are shown as K_D_. Results in Figs. 2A and 2B show that switching from IgG3 to IgG1, IgG2b or IgG2a was accompanied by a drop in functional affinity.

An alternative means to estimate relative functional affinity is the ELISA in which microtiter plate wells are coated with an excess of native γdPGA and antibody binding is determined via standard ELISA techniques. The results (Fig. 2C) showed that the amount of mAb required to produce a standard signal (OD_450_ = 2.0) with the IgG3 subclass was at least 15-fold less than was required for the subclass switch variants. These results confirm that switching from IgG3 to other subclasses produces a dramatic drop in functional affinity.

**Figure 3:**
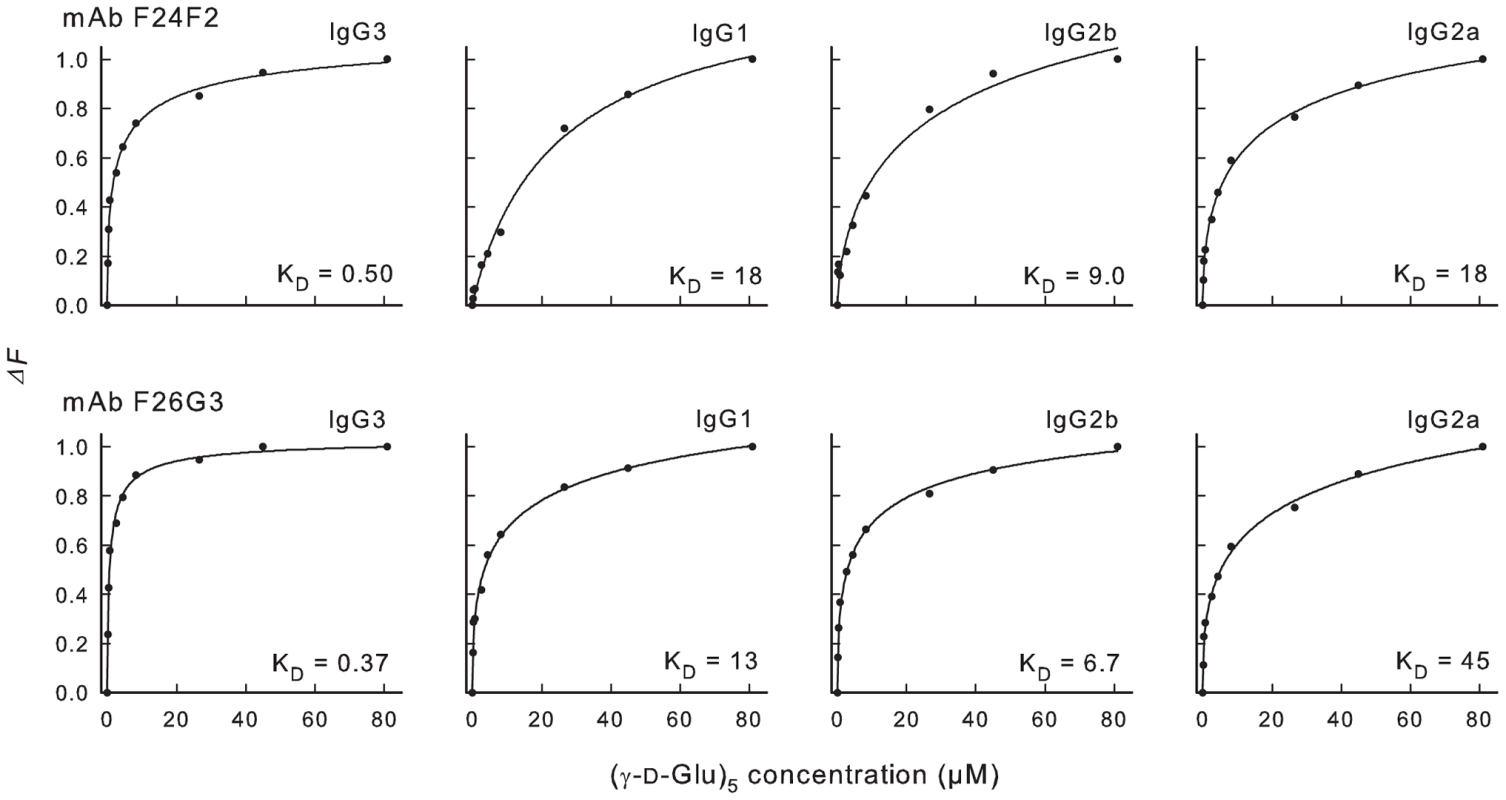
Assessment of binding of subclass switch families of mAbs F24F2 and F26G3 by fluorescence perturbation. Changes in mAb fluorescence (excitation wavelength = 284 nm; emission wavelength = 341 nm) are reported for parental F24F2 or F26G3 IgG3 and subclass switch variants upon addition of increasing amounts of synthetic (γ-d-Glu)_5_. The solid lines are hyperbolic fits of the data to the equation: y = (ax^b^)/(c^b^+x^b^), where c is the apparent dissociation constant (K_D_) in µM.

Analysis of mAb binding via surface plasmon resonance used chips that were coated with γdPGA oligomers of 25 residues. Experiments done under these conditions will allow for bivalent binding and therefore only measure functional affinity [25]. As a consequence, a second experiment was done in which fluid phase binding was assessed using (γ-d-Glu)_5_. A 5-mer peptide interacts with γdPGA mAbs via a monovalent binding process and measures intrinsic affinity [14], [23]–[25]. Intrinsic affinity is the interaction between one antibody paratope (binding site) and one epitope (antigenic determinant) [24]. Intrinsic affinity was measured using the change in intrinsic fluorescence (fluorescence perturbation, *ΔF*) of the mAb when incubated with increasing amounts of synthetic (γ-d-Glu)_5_. The results (Fig. 3) showed that the loss of functional affinity in switching from IgG3 to other subclasses shown in Fig. 2 is reflected in a loss of intrinsic affinity (18–120 fold loss relative to the parent IgG3). Indeed, the loss in intrinsic affinity with subclass switching was several-fold greater than the loss in functional affinity.

### Contribution of IgG heavy chain domains to protection and mAb binding

**Figure 4:**
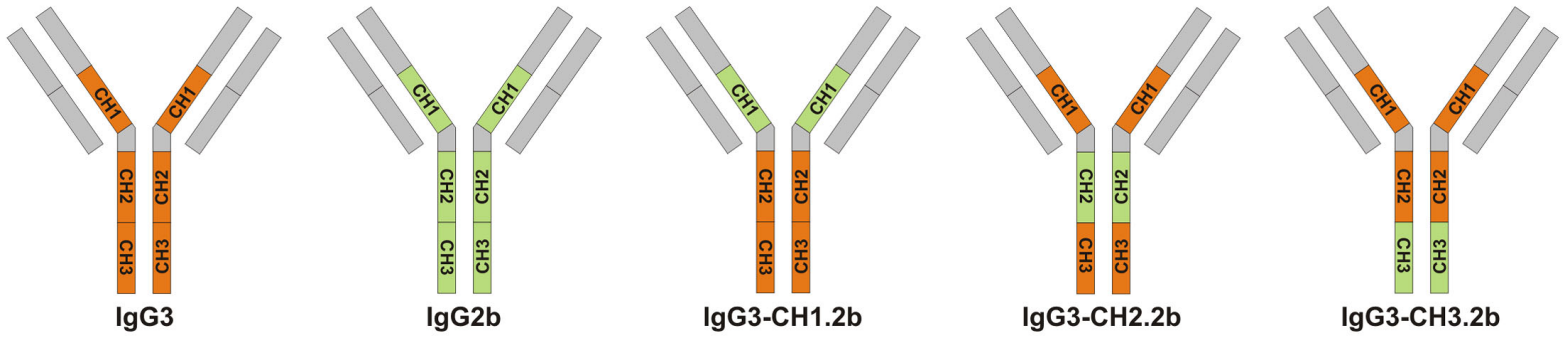
Nomenclature and construction of heavy-chain hybrids of mAb F26G3. The CH1, CH2 and CH3 domains of IgG3 mAb were replaced with the respective domains of the IgG2b subclass switch variant of mAb F26G3.

Results in Figs. 1, 2 and 3 indicated that some portion of the murine IgG heavy chain constant region influences both affinity for γdPGA and the ability of an antibody to protect. The constant region of the murine IgG heavy chain consists of the hinge region and three constant region domains: CH1, CH2 and CH3. A series of experiments was done in an effort to identify the portion of the heavy chain that contributes to the affinity and protective activity of the IgG3 γdPGA mAbs. To this end, hybrid antibodies were constructed that contained the framework of the IgG3 heavy chain substituted with the CH1, CH2 and CH3 domains of the low-affinity, non-protective F26G3 IgG2b subclass switch variant. A schematic that illustrates the composition and nomenclature of the various hybrid antibodies is shown in Fig. 4. The protective and binding properties of the hybrid mAbs were then evaluated in the same manner as described for the subclass switch variants.

**Figure 5:**
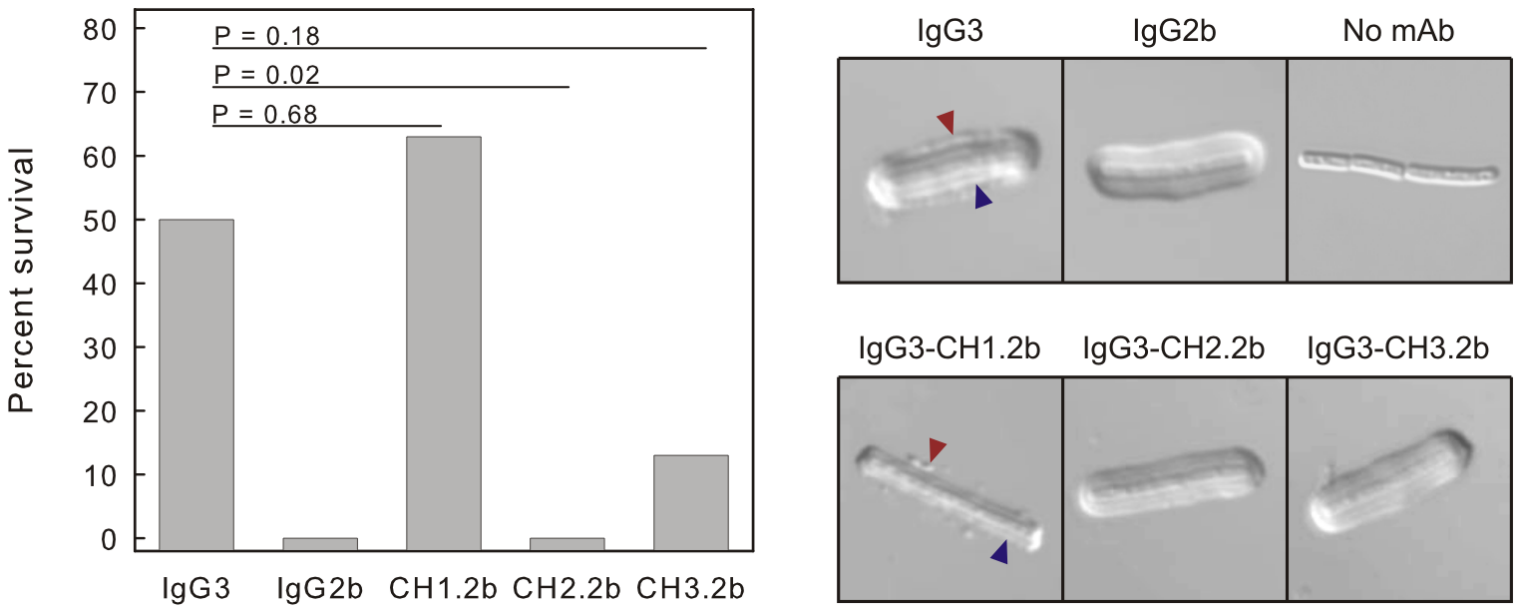
Protection and capsule reactivity of heavy-chain hybrids of IgG3/IgG2b (IgG3-CH1.2b, IgG3-CH2.2b, and IgG3-CH3.2b). See Fig. 4 for nomenclature. Left - Mice (n = 8/group) were treated with 800 µg of mAb and challenged 18 h later with 10 LD_50_ of *B. anthracis* (Ames) spores. Percent survival was evaluated using the Fisher exact test compared to the IgG3 treatment group. Right – Capsule reaction produced by incubation of *B. anthracis* vegetative cells with mAb F26G3 or the indicated hybrids of IgG3/IgG2b (75 µg/ml). Note reactivity of the IgG3 and IgG3-CH1.2b at both the outer edge (blue arrow) and an inner layer near the cell wall (red arrow).

Passive protection was assessed using a dose of 800 µg/mouse. This dose was chosen because it fell slightly below the maximal level of protection produced by the 1000 and 2000 µg doses of the F26G3 IgG3 and would be sensitive to a loss of protection produced by swapping of domains from the non-protective IgG2b. The results (Fig. 5) showed a high level of protection following treatment with the parent IgG3 or an IgG3 that contained the CH1 domain of the IgG2b. There was a significant (*P* = 0.02) loss of protection if the IgG3 contained the CH2 domain of the IgG2b.

Capsular quellung reactions were determined for the parent F26G3 IgG3 or hybrid IgG3 mAbs that contained each of the heavy region constant domains from the IgG2b. The results (Fig. 5) showed rim reactions or binding patterns that resemble a bead of pearls for the IgG3 and the IgG3 hybrid mAb containing CH1 from IgG2b (IgG3-CH1.2b). Binding to an inner layer of the capsule near the cell wall was also evident with the IgG3 hybrid mAb containing the CH1 domain from IgG2b. In contrast, hybrid IgG3 substituted with either the CH2 (IgG3-CH2.2b) or CH3 (IgG3-CH3.2b) domains of the IgG2b produced a puffy reaction that was identical to capsule reactions produced by the non-protective IgG1, IgG2b and IgG2a subclass switch variants.

**Figure 6:**
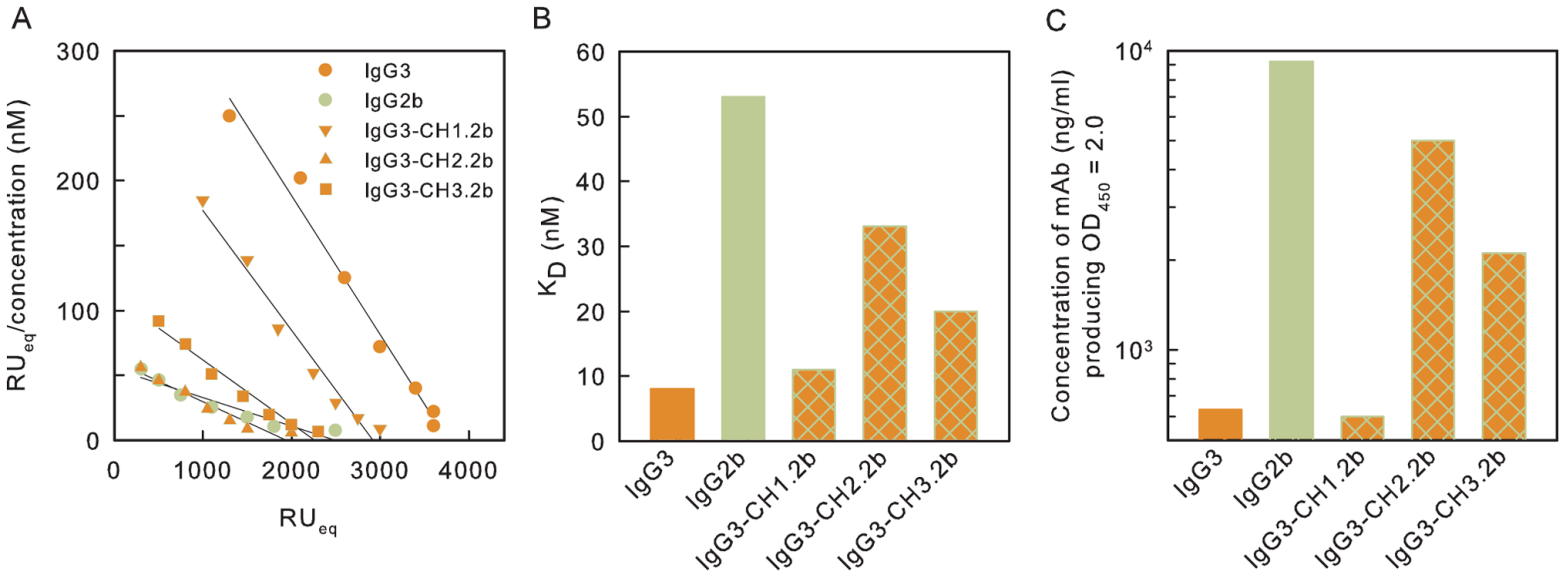
Binding behavior of parental and heavy-chain domain hybrid mAbs. A – Scatchard plots for determination of affinities from equilibrium binding by SPR. B – Dissociation constants (K_D_ in nM) for each mAb calculated from Panel A. C - Binding by ELISA. Results are reported as the concentration of each mAb (ng/ml) that produced an OD_450_ = 2.0 in a standard ELISA format.

Binding activities of the parent F26G3 IgG3 and the hybrid mAbs engineered with the CH1, CH2 or CH3 domains from IgG2b were assessed via surface plasmon resonance. The Scatchard plot is shown in Fig. 6A. There was a clear hierarchy of dissociation constants (K_D_) for the antibodies with IgG3∼IgG3-CH1.2b<IgG3-CH3.2b<IgG3-CH2.2b<IgG2b (Fig. 6B).

Finally, binding behavior of the parental and heavy-chain hybrid antibodies was assessed by ELISA. Microtiter plates were coated with γdPGA and incubated with serial dilutions of each mAb. The data are reported as the concentration of each mAb that produced an OD_450_ = 2.0. The results directly tracked results from the Scatchard analysis with the amounts of mAb produced to reach an endpoint: IgG3 = IgG3-CH1.2b<<IgG3-CH3.2b<IgG3-CH2.2b<IgG2b (Fig. 6C).

### Contribution of charge of CH domains to binding activity

The differences in charges between the various CH domains might contribute to the binding activity; polyglutamic acids have considerable negative charges (−1 per residue). As a consequence, we calculated the electrostatic potentials of CH1, hinge, CH2, and CH3 of each murine IgG subclass as well as the net charge of the full molecules. The results of the calculations are reported in Table 1.

**Table 1:** Net charge of each murine IgG subclass and domain at pH 7.4^a^.

Subclass	Heavy chain domain				
	CH1	Hinge	CH2	CH3	Total
IgG3	3.0	1	2.2	−1.7	4.5
IgG1	2.1	0.8	−2.9	0.4	0.4
IgG2b	1	−0.1	−2.8	2.1	0.2
IgG2a	3	−0.1	−1.8	2.2	3.3

a Net charges were calculated assuming an environment of pH = 7.4 using amino acid sequences obtained from the ImMunoGeneTics online database (www.IMGT.org).

**Figure 7:**
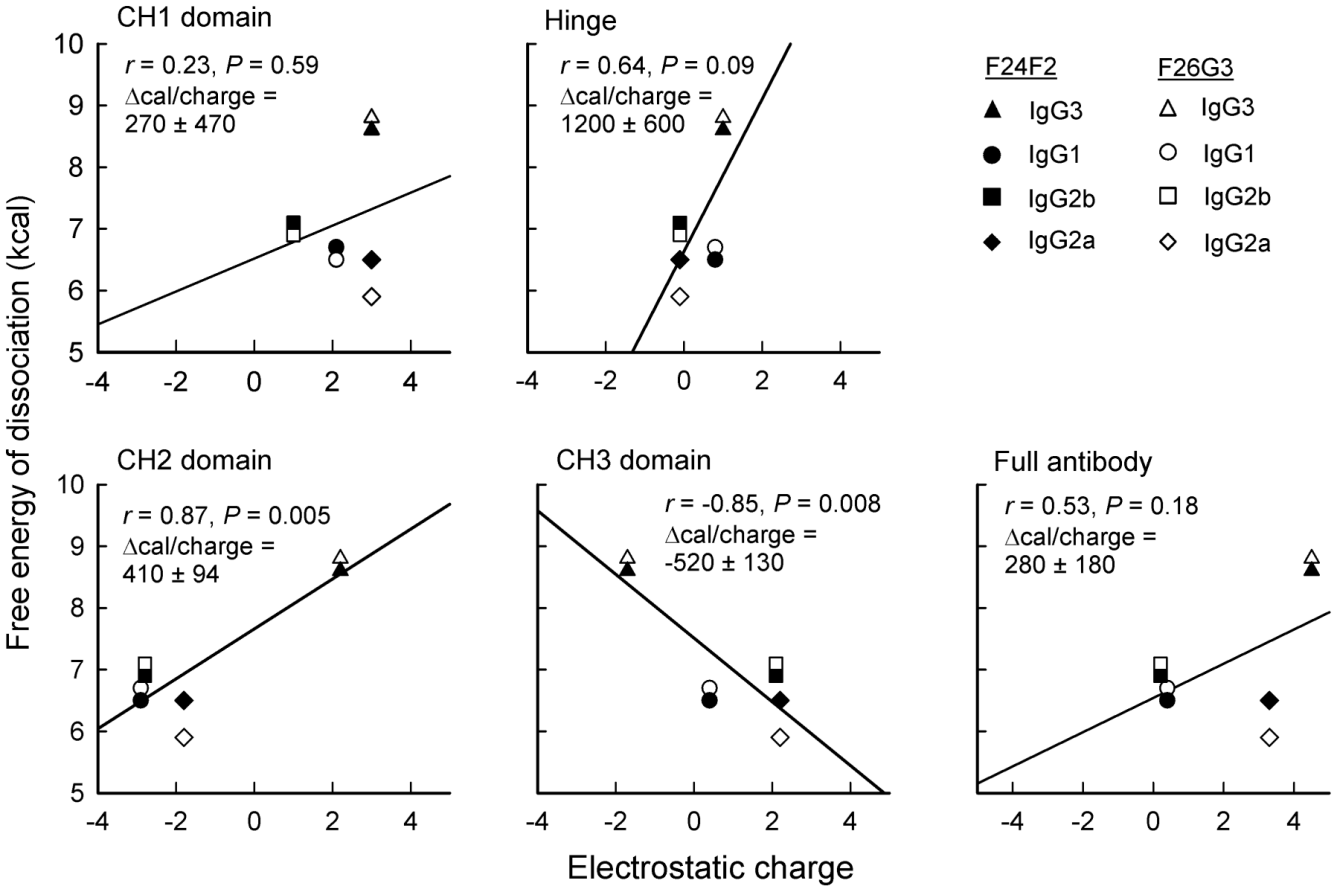
Correlation between charge of the CH1, hinge, CH2, CH3 and intact mAbs F24F2 and F26G3 of different IgG subclasses and the free energy of dissociation of each mAb. Free energy of dissociation was calculated from the K_D_ of each mAb as determined by fluorescence perturbation assay using a synthetic (γ-d-Glu)_5_ as the ligand (data from Fig. 3).

The K_D_ values of the F24F2 and F26G3 subclass switch variant mAbs determined by fluorescence perturbation assays (Fig. 3) were converted to the free energy of dissociation (ΔG_0_), plotted against the respective charge of CH1, CH2, CH3, the hinge region and the full antibody molecule of each subclass each mAb, and a correlation was determined (Fig. 7). There was no significant correlation between the net charge of the full antibody molecule and binding energy (*r* = 0.53; *P* = 0.18). Further analysis also showed no correlation between charge and binding energy for the CH1 (*r* = 0.23; *P* = 0.59) and the hinge regions (*r* = 0.64; *P* = 0.085). The two domains that comprise the Fc portion of the antibodies had higher but opposing correlation coefficients: CH2 (*r* = 0.87; *P* = 0.005) and CH3 (*r* = −0.85; *P* = 0.008).

## Discussion

Many bacteria are readily killed by phagocytic cells. As a consequence, pathogens may produce extracellular capsules that inhibit phagocytosis and killing. Mammalian hosts in turn produce antibodies that bind to capsules to facilitate phagocytosis or complement-mediated killing of extracellular bacteria. As a consequence, targeting capsules through active or passive immunization is a key strategy for control of many bacterial diseases. Immunity that targets bacterial capsules is, therefore, critically dependent on the interaction of antibodies with capsular antigens [26].

The immune responses to different capsular antigens share many features (reviewed in [26]). Most capsules are polysaccharide in composition and have identical repeating units composed of one to six monosaccharides. Most capsular polysaccharides lack T cell-dependent immunogenicity, do not induce a booster antibody response, and fail to produce protective serum antibody in infants and young children. Finally, the antibody response to immunization with capsular polysaccharides is highly restricted; mice produce primarily IgG3 [27], [28] and humans produce primarily IgG2 (reviewed in [29]). This restriction has led to speculation as to the advantage to the host of murine IgG3 or human IgG2 over other IgG subclasses in interaction with capsular polysaccharides [22], [30].

The *B. anthracis* capsule is composed of a polypeptide (γdPGA) rather than a polysaccharide. However, γdPGA has many structural and immunological properties in common with capsular polysaccharides described above, including being a T-independent antigen [8], [11]. Given the similarities between γdPGA and capsular polysaccharides, a major goal of this study was to evaluate the nature of the interaction between γdPGA and its cognate antibodies and to place these results in the larger context of antibody binding to capsular polysaccharides.

Results from the present study identify a key role for antibody constant regions in i) intrinsic affinity, ii) functional affinity, iii) patterns of binding to the capsule and iv) protection in a murine model of pulmonary anthrax. Construction of hybrid antibodies in which heavy-chain domains of the protective IgG3 mAb were replaced with their respective domains from a low-affinity and non-protective IgG2b mAb showed that the CH1 domain did not contribute to either protection or affinity. The primary contribution of heavy chain domains to protection and affinity was found to be in the CH2 domain with a lesser contribution by the CH3 domain.

The classical explanation for binding between antigen and antibody is that of complementarity between the antibody binding site (paratope) and a monovalent ligand (epitope). CH domains contribute to functional affinity due to the polyvalent nature of intact immunoglobulins. However, this classical view has been challenged in two series of studies of antibody binding to polysaccharide antigens and a recent report of antibody binding to HIV-1 [31]. The binding of mAbs to γdPGA adds to this body of evidence for a role of CH domains in antibody binding, but the mechanism appears to be fundamentally different from previous reports.

In one example, Casadevall and colleagues examined a subclass switch family of mAbs against the glucuronoxylomannan (GXM) capsule of *C. neoformans*. GXM IgG2b, IgG2a, and IgG1 mAbs were protective in a murine model of cryptococcosis, whereas IgG3 mAbs provided no protection [32], [33]. Evaluation of thermodynamic and kinetic parameters of mAb binding found the non-protective IgG3 mAb to have the lowest binding affinity within the set [34]. The binding properties of the parent antibodies were largely reflected in the binding of Fab fragments, suggesting that structural differences in the CH1 domain are responsible for the differences in affinity constants.

Our studies of γdPGA mAbs differ from those of GXM mAbs in two fundamental respects. First, γdPGA mAbs of the IgG3 subclass showed the greatest affinity and highest level of protection; IgG3 mAbs binding to GXM showed the lowest affinity and the lowest level of protection. Second, switching of domains from the low affinity, non-protective IgG2b variant of γdPGA mAb F26G3 into the IgG3 framework localized both the protective effect and high affinity binding to the CH2–CH3 domains rather than the key role played by CH1 in binding of GXM mAbs.

In the second example, seminal studies by Greenspan and colleagues found that IgG3 mAbs reactive with the *N*-acetyl-glucosamine (GlcNAc) residues of streptococcal group A polysaccharide had a higher functional affinity than variable region-identical subclass switch IgG1 and IgG2b mAbs [35]–[37]. The high functional affinity of IgG3 antibodies was due to Fc-dependent cooperative interactions. Enhanced functional affinity required an intact Fc region, identifying CH2–CH3 as the region of the IgG3 heavy chain required for cooperative interaction.

Binding of mAbs to γdPGA was similar to binding of GlcNAc mAbs in two key respects. First, IgG3 mAbs showed the highest functional affinity relative to other subclasses. Second, the enhanced functional affinity of both the γdPGA and GlcNAc IgG3 antibodies was due to the CH2–CH3 domains. Indeed, the available evidence (Fig. 6) suggests that the CH2 domain plays a larger role in affinity than CH3. However, there is a key fundamental difference between the binding activities of γdPGA and GlcNAc mAbs that suggests different mechanisms. In the case of the GlcNAc mAbs, the binding advantage of the IgG3 subclass was attributed to enhanced functional affinity that results from an increased valence due to Fc cooperativity. In contrast, the binding advantage of the intact IgG3 γdPGA mAb (functional affinity determined via SPR or ELISA, Fig. 2) was directly reflected in the higher intrinsic affinity (fluorescence perturbation with a 5-mer peptide) of the mAb relative to other subclasses (Fig. 3). Indeed, the differences in intrinsic affinity of mAbs of different subclasses were considerably greater than the respective differences in functional affinity. These results indicate that cooperative binding alone cannot explain the contribution of heavy chain domains to affinity of γdPGA mAbs.

A third potential contribution by CH domains to antibody binding was suggested by Morelock et al. [38] who found that differences in the functional affinity of a family of murine/human chimeric mAbs (huIgG1>huIgG4>huIgG2) reflected the flexibility of the hinge region (IgG1>IgG4>IgG2; [39]). In contrast, our studies of variation in the functional affinity of γdPGA mAbs (IgG3>IgG1>IgG2b∼IgG2a) did not reflect the hierarchy in flexibility of the murine IgG (IgG2b>IgG2a>IgG3>IgG1; [39]).

Our studies support previous arguments that the heavy chain constant regions of IgG3 contribute to binding to antigen [35], [40]–[42]. The evolutionary or adaptive value for development of enhanced binding by IgG3 is not known. As noted by Greenspan and Cooper [42], the IgG3 C region gene is the most highly conserved of the Ig heavy chain C region genes among inbred mice [43], [44]. Involvement of CH domains in binding (positive or negative effects) may be a general characteristic of antibodies to antigens with repeating subunits such as those in GXM, GlcNAc or γdPGA. Notably, GXM and γdPGA are both T-independent antigens.

Production and evaluation of CH domain hybrids found the greatest contribution of the IgG3 heavy chain to antibody binding to be in CH2 in two distinct assay systems – surface plasmon resonance and ELISA (Fig. 6). The available data do not provide insights into a mechanism by which CH2 can contribute to affinity. One possibility is glycosylation. CH2 is heavily glycosylated; however, our preliminary studies have found that deglycosylation of IgG3 has no effect on functional affinity (unpublished results).

A second possible explanation for the contribution of CH domains to antibody binding is that of charge. Charge could contribute to binding by direct electrostatic or coulombic attraction between the antibody and the negatively charged γdPGA. However, the measured binding energy of each antibody varied little with the net charge of the whole antibody (Fig. 7). Further dissection of the electrostatic charges that are present on individual antibody domains showed no correlation between electrostatic charge of the CH1 and hinge. We found a significant correlation between electrostatic charge and the respective binding energies when the CH2 and CH3 were analysed; however, the correlation is opposite for the two domains. In unpublished studies, we have found that neither mAb F24F2 or F26G3 showed any appreciable binding to poly-l-glutamic acid. Because poly-l-glutamic acid carries the same charge as γdPGA, it is unlikely that simple coulombic interactions are major contributors to the differences in binding energy seen among the four subclasses of murine IgG. However, our results do not exclude the possibility that charge in CH2 influences conformation or permissivity in the antibody paratope.

An alternative mechanism by which the C domain could influence antibody binding is by imposing structural constraints on the V region that alter the chemical and/or electronic environment within the antibody paratope or the ability to undergo a conformational change on epitope binding [45], [46]. For example, Janda et al. recently reported that the murine IgG constant regions influenced the energy landscape of the variable region [46]. Our data do not provide information on possible effects of CH on the antibody paratope, but the results map the effects of CH on binding to the CH2/CH3 regions.

Considerable effort has gone into mutagenesis of antibody variable regions to produce mAbs with enhanced affinity. Enhancement of affinity may improve the therapeutic efficacy of many mAbs or enhance the sensitivity of diagnostic tests in which antibodies are the test reagent. Identification of a region in CH2 that contributes to affinity may allow for affinity-enhancement of γdPGA mAbs of the IgG1, IgG2b or IgG2a subclasses if the active regions of the IgG3 were swapped into mAbs of other subclasses. More interesting is the possibility that swapping the affinity-enhancing region of IgG3 CH2 into other mAbs with specificity for polysaccharides or proteins could produce a significant affinity enhancement. If this were the case, a single antibody engineering solution might improve antibody performance without the need for affinity-enhancement of each mAb variable region.

Finally, limitations to the experimental design should be noted. The experimental approach we used was that of loss of function, which showed a striking congruence between loss of protection and loss of binding activity as shown by i) interactions with the capsular matrix and ii) binding activity via SPR and ELISA. Future studies will examine gain of function and produce hybrid mAbs in which smaller segments of CH2 from IgG2b are exchanged into IgG3 (and vice versa) to further refine the structural requirements for affinity enhancement by the IgG3 heavy chain. We also recognize that the protective activity of the IgG3 mAbs likely includes a mosaic of factors of which affinity is one critical element. The biological activities of IgG antibodies are heavily influenced by the distinct biological activities of each subclass and the engagement of different Fc receptors by antibodies of different subclasses. However, the biological activities of IgG3 do not distinguish themselves in a manner that would suggest greater protective activity relative to the other subclasses. For example, mouse IgG3 has a segmental flexibility that is slightly greater than IgG1 but less than IgG2a or IgG2b [39]. Like IgG2a and IgG2b, mouse IgG3 is a potent activator of the classical complement system [39], [47]. Mouse IgG3 binds to mouse FcγRI with low affinity but shows limited or no binding to mouse FcγRIIB, FcγRIII or FcγRIV [48]–[50].

In summary, our studies identify important parameters for antibody-mediated protection that targets the *B. anthracis* capsule. The CH2 domain of murine IgG3 is critical for protection, intrinsic affinity and functional affinity. The involvement of the CH2 domain in affinity was found to be a novel mechanism by which the IgG heavy chain contributes to antibody affinity and protection. Finally, these results support arguments that the influence of antibody heavy chain regions on antibody binding should be an important consideration in the development of genetically engineered antibodies for therapeutic use [45], [51]. Optimization of mAbs as therapeutic agents will require a better understanding of the interactions between antibody constant and variable regions and the effects of such interactions on antigen binding.

## Materials and Methods

### Ethics statement

This study was carried out in accordance with recommendations in the Guide for the Care and Use of Laboratory Animals of the National Institutes of Health. These specific protocols were approved by the University of New Mexico Institutional Animal Care and Use Committee (Animal Welfare Assurance Number AS3350-01).

### Subclass-switch families of mAbs

Full subclass-switch families (IgG3→IgG1→IgG2b→IgG2a) were generated from parental F24F2 and F26G3 anti-γdPGA IgG3 mAbs by the sequential sublining method of Spira et al. [18]. Both mAbs are V_H_ IMGT subgroup IGVH10S2*02 and V_L_ subgroup IGKV1-135*01; however, the mAbs differ in the JH and JL regions [14]. Accession numbers for the V sequences are available online at the GenBank database (accession numbers EF030730 and EF030736 for VH and VL of F24F2, respectively, and EF030731 and EF030737 for VH and VL of F26G3). Switching from one subclass to another follows the germline order of heavy chain exons (γ3, γ1, γ2b, γ2a). The procedure was done in a sequential manner to obtain hybridomas that secrete mAbs of the IgG1, IgG2b and IgG2a subclasses.

Once hybridoma cell lines were established, total mRNA was isolated from cells using a Straight A's mRNA Isolation System (Novagen Inc., Madison, WI) and cDNA synthesis using a First Strand cDNA Synthesis Kit (Novagen Inc.) with poly-T primers. cDNA was PCR amplified using 5′ end primers specific for the heavy and light chain variable region of F24F2 and F26G3. PCR products were sequenced by Nevada Genomics Center to verify identical variable region sequence and correct subclass sequence.

### Generation of CH1, CH2 and CH3 domain hybrid mAbs

CH domain hybrid mAbs were generated with an overlap PCR technique using F26G3 IgG3 and IgG2b template DNA. PCR primers were designed with overlapping sequences and are listed in Table S1. Platinum PCR SuperMix High Fidelity (Invitrogen, Carlsbad, CA) was used for all PCR reactions.

Briefly, for generation of F26G3 IgG3-CH1.2b mAb the following PCR reactions were performed, i) PCR1 forward and reverse primers were combined with F26G3 IgG3 template, ii) PCR2 primers were combined with F26G3 IgG2b template, and iii) the resulting PCR1 and PCR2 fragments were purified, combined and amplified with PCR3 primers to generate a full length IgG3-CH1.2b PCR fragment. This fragment was TA cloned to pGEM-T easy vector and transformed to NEB 5-alpha competent *E. coli* cells (New England BioLabs Inc., Ipswich, MA). Positive clones were sequenced at the Nevada Genomics Center. The F26G3 IgG3-CH1.2b sequence was cleaved from the pGEM vector with XbaI and AgeI restriction enzymes (New England BioLabs Inc., Ipswich, MA) and cloned to pcDNA3.3-TOPO expression vector (Invitrogen/Life Technologies, Grand Island, NY) that was digested with the same enzymes. After transformation to *E. coli* cells, positive clones were purified and sequenced. The additional heavy chain hybrid mAbs (IgG3-CH2.2b and IgG3-CH3.2b) were cloned in a similar fashion. F26G3 light chain was PCR amplified with primers (Table S1) and cloned to pOptiVEC-TOPO vector (Invitrogen) according to product manual. Each heavy chain hybrid plasmid was separately transfected with the F26G3 light chain pOptiVEC-TOPO plasmid into a DG44 cell line, and the hybrid mAbs were produced according to the OptiCHO Antibody Express Kit product manual (Invitrogen). All hybrid CH mAbs were isolated from growth medium by affinity chromatography on protein A (GE Healthcare, Piscataway, NJ).

### Immunochemical assays

γdPGA for immunochemical assays was isolated as described [14] from culture filtrates of *B. licheniformis* strain 9945 that was grown on Medium E that contained 2 mM MnCl_2_•4 H_2_O to stimulate maximal production of PGA in the D isoform [52]. An acid hydrolysate of the purified γdPGA exhibited a specific optical rotation (−25.2°) indicating that ∼84% of the glutamic acid was the D isomer.

#### Capsule reactions

Formalin-killed *B. anthracis* Ames bacilli were generated at the University of New Mexico Health Sciences Center. The Ames strain was originally obtained from the U.S. Army Medical Research Institute of Infectious Diseases (USAMRIID, Frederick, MD). Capsular “quellung” – type reactions were determined by incubating formalin-killed *B. anthracis* Ames bacilli with various mAbs (75 µg/ml) for 10 min at 37°C. Capsular reactions were evaluated by differential interference contrast (DIC) microscopy.

#### ELISA

Polystyrene plates (Thermo, Waltham, MA) were coated for 5 h with 0.005% w/v Poly-l-Lysine (Sigma, St. Louis, MO) diluted in PBS. Plates were washed with PBS and incubated overnight with γdPGA (2 µg/ml) in PBS. Plates were washed with PBS+0.05% Tween 20 and blocked with PBS+0.5% Tween 20, 5% milk (blocking solution) at 37°C for 1.5 h. mAbs were added onto plates by serial diluting (100 µg/ml to 20 ng/ml) with blocking solution and incubated for 1.5 h. mAb binding was detected with peroxidase-conjugated goat anti-mouse kappa chain antibody (Southern Biotech, Birmingham, AL) followed by addition of TMB peroxidase substrate (KPL, Gaithersburg, MD). The enzymatic reaction was stopped with a 5% solution of o-phosphoric acid and the enzyme product was determined by measuring absorbance at 450 nm. Results are reported as the concentration of each mAb that produced an OD_450_ = 2.0. The experiment was repeated three times with similar results.

#### Surface plasmon resonance (SPR)

Binding of mAbs to γdPGA was evaluated by SPR using a BIAcore X100. The running and sample buffer used for all experiments was HBS-EP+ buffer, pH 7.4, (10 mM HEPES, 150 mM NaCl, 3 mM EDTA, and 0.05% surfactant P20). For ligand preparation, γdPGA oligomers of 25 residues in length were synthesized as previously described [14]. γdPGA oligomers were biotinylated by standard amine coupling chemistry (Pierce, Rockford, IL) and purified by size exclusion chromatography (Pierce). Biotinylated peptides were immobilized onto a SA sensor chip (GE Healthcare) until immobilization levels of 50–100 response units (RU) were reached. A flow cell was left unmodified for reference subtraction.

To evaluate binding, mAb samples were diluted in HBS-EP+ and analyzed at several concentrations (5, 10, 21, 42, 83, 167, 333 nM). mAb at each specified concentration was injected over the modified chip surface for 3 min at 30 µl/min. The chip surface was regenerated between runs with a 1 min pulse of 4M MgCl_2_. Identical runs were done in triplicate for each antibody.

Antibody binding affinity was calculated from analysis of equilibrium binding as described [53]. In this analysis, RU_eq_ (equilibrium resonance units)/C (concentration of free protein) versus RU_eq_ yields a Scatchard plot with a slope of –K_A_. For comparison to fluorescence perturbation data, K_A_ was converted to K_D_ by taking the negative reciprocal value.

#### Fluorescence perturbation

Fluorescence perturbation analysis was used to measure mAb interaction with γdPGA in fluid as described [14]. All titrations were performed on a Spex 111 spectrofluorometer using photon counting. Binding was measured by adding small aliquots of synthesized 5-mer of γdPGA in PBS to a solution of each mAb (25 µg/ml) in PBS. The fluorescence intensity was measured using 284 nm excitation and 341 nm emission wavelengths and right angle geometry. Fluorescence data was corrected for excitation intensity. Five successive 10 s integration periods were averaged for each time point. The fluorescence yield was constant over this time period. Background fluorescence from buffer blank was subtracted from all emission spectra. The emission spectra of all solutions were corrected for dilution. Results are reported as *ΔF* vs. the concentration of added (γ-d-Glu)_5_ (µM) where *ΔF* = (F−F_o_)/(F_sat_−F_o_) with F_o_ = initial fluorescence intensity, F_sat_ = fluorescence intensity at saturation, and F = fluorescence intensity at the indicated isopeptide concentration.

Plots of *ΔF* (fluorescence) vs. γdPGA concentration were constructed. The K_D_ in molar units was determined from the plots as the γdPGA concentration at one-half *ΔF_max_* that was estimated by computer-aided fit to a hyperbolic binding isotherm (SigmaPlot, Systat Software Inc., San Jose, California).

### Calculations of mAb electrostatic potential and free energy of dissociation

The amino acid sequences of the CH1, hinge region, CH2, and CH3 of each murine IgG subclass were obtained from the ImMunoGeneTics online database (www.IMGT.org). These sequences were used to calculate the net charge of each murine IgG subclass as a whole, and the net charge of each antibody domain/hinge region separately. The net charges were calculated assuming an environment of pH 7.4. Net charge of each immunoglobulin domain was determined by use of PepCalc (Innovagen AB). The binding affinities (K_D_) of each murine IgG subclass of mAbs F24F2 and F26G3 were used to calculate the thermodynamic free energy of dissociation (ΔG_0_) using the equation: ΔG_0_ = −RTlnK_D_, where R is the gas constant (1.986 cal K^−1^ mol^−1^), T is the temperature (K), and K_D_ is the dissociation constant (M) of each antibody as determined by fluorescence perturbation (from Fig. 3). Graphs plotted net charge and the charge of each domain against ΔG_0_ for each antibody. Correlation was evaluated using the Pearson product-moment correlation coefficient (*r*).

### Murine model of pulmonary anthrax

BALB/c mice were treated intraperitoneally with mAbs diluted with DPBS or with vehicle (DPBS) alone as a control. The mice were infected 18 h after mAb treatment by intratracheal challenge with 10^4^ (10 LD_50_) *B. anthracis* Ames strain spores in 50 µl. The actual number of spores deposited in the lung was determined for each experiment by sacrificing three mice following infection, homogenizing the lungs in 1 ml of DPBS, culturing serial dilutions on sheep blood agar plates, and averaging the number of colony forming units. Survival and clinical signs were monitored daily for 14 days post-infection. Percent survival was compared to the vehicle-treated control by the Fisher exact test (SigmaStat 3.5, Systat Software, Inc.).

## Supporting Information

Table S1 PCR primers used to produce heavy chain domain hybrids and light chain sequences.
(DOC)
