# Supplementary material for: IgG Subclass and Heavy Chain Domains Contribute to Binding and Protection by mAbs to the Poly γ-D-glutamic Acid Capsular Antigen of Bacillus anthracis
Source: PLoS Pathog. 2013 Apr 18;9(4):e1003306. doi: 10.1371/journal.ppat.1003306 (PMC3630167; doi:10.1371/journal.ppat.1003306)
Supplement: Table S1 — PCR primers used to produce heavy chain domain hybrids and light chain sequences. (DOC) [file ppat.1003306.s001.doc]

| Table S1. PCR primers used to produce heavy chain domain hybrids and light chain sequences | | |
| --- | --- | --- |
| Constant region constructs | Forward Primer (5’ – 3’) | Reverse Primer (5’ – 3’) |
| IgG3-CH1.2b |  |  |
| PCR1 | gcgtctagaaccatggacttcggg | cttgggtattctaggctcaagttttttgtccac |
| PCR2 | gtggacaaaaaacttgagcctagaatacccaag | ctaccggtagctcatttaccagggga |
| PCR3 | gcgtctagaaccatggacttcggg | ctaccggtagctcatttaccagggga |
| IgG3-CH2.2b |  |  |
| PCR1 | gcgtctagaaccatggacttcggg | gtccaccctcgaggttaggaggtgggcatgaagaacc |
| PCR2 | ggttcttcatgcccacctcctaacctcgagggtggac | cttgaggtgtctgggctctccctttaatttttgagatgg |
| PCR3 | gcgtctagaaccatggacttcggg | cttgaggtgtctgggctctccctttaatttttgagatgg |
| PCR4 | ccatctcaaaaattaaagggagagcccagacacctcaag | ctaccggtagctcatttaccagggga |
| PCR5 | gcgtctagaaccatggacttcggg | ctaccggtagctcatttaccagggga |
| IgG3-CH3.2b |  |  |
| PCR1 | gcgtctagaaccatggacttcggg | agctctgactagccctttgggttttgagatg |
| PCR2 | catctcaaaacccaaagggctagtcagagct | ctaccggtagctcatttaccagggga |
| PCR3 | gcgtctagaaccatggacttcggg | ctaccggtagctcatttaccagggga |
| F26G3 light chain |  |  |
| PCR1 | acgaagcttaccatgaagtttccttct | ttctctagaatcaacactcattcctg |
